# VirF-Independent Regulation of *Shigella virB* Transcription is Mediated by the Small RNA RyhB

**DOI:** 10.1371/journal.pone.0038592

**Published:** 2012-06-11

**Authors:** William H. Broach, Nicholas Egan, Helen J. Wing, Shelley M. Payne, Erin R. Murphy

**Affiliations:** 1 Department of Biological Sciences, Molecular and Cellular Biology Program, Ohio University, Athens, Ohio, United States of America; 2 School of Life Sciences, University of Nevada, Las Vegas, Las Vegas, Nevada, United States of America; 3 Section of Molecular Genetics and Microbiology, The University of Texas at Austin, Austin, Texas, United States of America; 4 Department of Biomedical Sciences, Ohio University Heritage College of Osteopathic Medicine, Athens, Ohio, United States of America; University of Padova, Medical School, Italy

## Abstract

Infection of the human host by *Shigella* species requires the coordinated production of specific *Shigella* virulence factors, a process mediated largely by the VirF/VirB regulatory cascade. VirF promotes the transcription of *virB*, a gene encoding the transcriptional activator of several virulence-associated genes. This study reveals that transcription of *virB* is also regulated by the small RNA RyhB, and importantly, that this regulation is not achieved indirectly via modulation of VirF activity. These data are the first to demonstrate that the regulation of *virB* transcription can be uncoupled from the master regulator VirF. It is also established that efficient RyhB-dependent regulation of transcription is facilitated by specific nucleic acid sequences within *virB*. This study not only reveals RyhB-dependent regulation of *virB* transcription as a novel point of control in the central regulatory circuit modulating *Shigella* virulence, but also highlights the versatility of RyhB in controlling bacterial gene expression.

## Introduction

Shigellosis, a severe diarrheal disease caused by members of the *Shigella* genus (*S. dysenteriae, S. flexneri, S. sonnei* and *S. boydii*) remains a worldwide health concern with a conservative estimate of 165 million cases resulting in over one million deaths each year [1]. Following ingestion and transit through the gastrointestinal tract of the host, *Shigella* invade cells of the colonic epithelium, replicate within the cytoplasm of the infected cell and spread to neighboring epithelial cells via actin based motility [2], [3]. These virulence-associated processes are mediated by the coordinated production and activity of several *Shigella* virulence factors.

**Figure 1:**
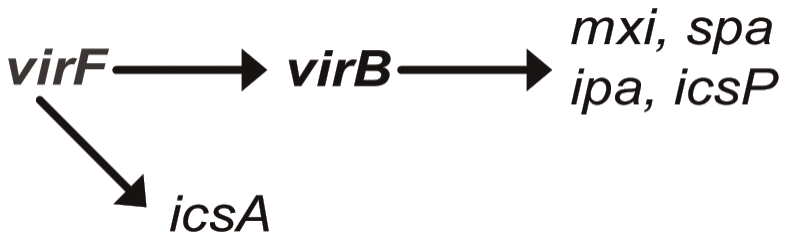
Schematic of the VirF/VirB cascade. VirF is produced under permissive conditions and positively regulates the expression of *icsA* and *virB.* VirB in turn activates the expression of many virulence-associated genes including those encoding components of the Type Three Secretion System and associated effectors (*mxi, spa* and *ipa*) as well as IcsP, the protease specific for the actin based motility protein IcsA.

The complex and coordinated regulation of *Shigella* virulence gene expression is accomplished largely via the activity of two transcriptional activators, VirF and VirB [4], [5]. Activity of the VirF/VirB regulatory cascade, and thus *Shigella* virulence, is modulated in response to specific environmental signals such as changes in temperature, pH and osmolarity, as well as carbon source and iron availability [1], [6], [7], [8]. In response to precise environmental conditions, specifically those encountered within the host, VirF is produced and directly activates the transcription of two virulence-associated genes, *icsA* and *virB*. IcsA facilitates intracellular spread of *Shigella* by mediating actin-based motility [9], while VirB directly promotes the expression of multiple virulence-associated genes (Figure 1). The VirB regulon includes genes encoding components of the Type Three Secretion System (TTSS), *icsP* encoding the protease that modulates IcsA activity, and *mxiE* encoding a transcriptional activator of additional virulence associated genes [1], [3], [4], [10], [11]. As a central pathway controlling the coordinated expression of several virulence-associated genes, any factor that influences the production or activity of VirF or VirB consequently impacts *Shigella* virulence.

Expression of genes within the VirF and VirB regulons appears to be coupled: factors that impact the VirF regulon, also impact the VirB regulon. The coupled expression of genes within the VirF and VirB regulons results from the fact that both transcriptional and post-transcriptional regulation of *virB* is mediated by factors or environmental conditions that also modulate the expression of *virF*. Specifically, transcriptional regulation of *virB* is mediated by VirF itself, or by H-NS and IHF, proteins known to influence *virF* expression [5], [12], [13]. Post-transcriptional regulation of *virB* expression, via destabilization of the *virB* mRNA, is mediated in response to changes in environmental pH, osmolarity and temperature [14], [15], [16], conditions that also influence expression of *virF* [12], [17], [18], [19]. No study to date has identified a regulatory factor that controls the transcription of genes within the VirB regulon, such as *icsP* or *mxiE,* without also regulating *icsA*, a gene that lies solely within the VirF regulon (Figure 1).

Of the environmental conditions known to modulate *Shigella* virulence factors via regulation of the VirF/VirB cascade, the impact of iron-availability is the least well characterized to date. Under conditions of iron-limitation, the small RNA RyhB is produced and functions to inhibit *virB* expression, as evidenced by a reduction in the steady state level of *virB* mRNA [6], a decrease in the production of proteins within the VirB regulon [6], [20] and an inhibition of epithelial cell invasion by the bacterium [6]. While it is clear that RyhB functions to reduce the steady state level of *virB* mRNA and inhibit VirB activity [6], it remains unclear whether RyhB-dependent regulation of *virB* mRNA is achieved through the modulation of VirF activity, whether the regulation occurs in a VirF-independent manner at the level of *virB* transcription, or whether the *virB* message is simply targeted for accelerated degradation in the presence of RyhB. A greater understanding of the RyhB-dependent regulation of *virB* expression is needed to improve our knowledge of the VirF/VirB cascade, which controls virulence gene expression in *Shigella.*

## Materials and Methods

### Growth Conditions

Bacterial strains and plasmids used in this study are shown in Table 1. *Escherichia coli* was cultured in Luria-Bertani (LB) broth (1% tryptone, 0.5% yeast extract and 1% NaCl) or on LB agar plates at 37°C. *Shigella dysenteriae* was cultured in LB broth or on tryptic soy broth (Becton, Dickenson and Company, Sparks, MD) agar plates containing 0.01% (wt/vol) Congo red dye (ISC Bioexpress, Kaysville, UT) at 37°C.

**Table 1:** Bacterial strains and plasmids.

Strain or Plasmid	Description	Source
***Escherichia coli*** ** strains**		
DH5α		Life Technologies
DH5α(λpir)		[39]
***Shigella*** ** strains**		
O-4576S1	Wild-type *S. dysenteriae*	[40]
ND100 (wild-type)	Spontaneous Streptomycin resistant mutant of O-4576S1	[6]
ND100*ryhB*	*ryhB* deletion in ND100	[6]
ND100*ryhB*alt*virB*	Site-specific mutation within *virB* of ND100*rhyB*	This study
2457T	Wild-type *S. flexneri*	[41]
**Plasmids**		
pCVD442N2	Suicide vector	[42]
pERM124	Altered *virB* construct in pCVD442N2	This study
pQE2	Expression vector	QIAGEN
p*ryhB*	*ryhB* in pQE-2	[6]
pComp*ryhB*	Compensatory *ryhB* in pQE-2	This study
pRW50	*lacZ* reporter plasmid	[24]
P*icsA-lacZ*	*icsA* promoter region transcriptionally fused to *lacZ* in pSRG12	Gift from M. Goldberg.This study
P*icsP-lacZ* (previously pHJW20)	*icsP* promoter region transcriptionally fused to *lacZ*	[27]

### Oligonucleotide Primers and Probes

The name, nucleic acid sequence and function of each oligonucleotide primer and probe used in this study are presented in Table 2.

**Table 2:** Primers and Probes.

	Sequence	Use in study
**Primers**		
virB2-for	TCCAATCGCGTCAGAACTTAACT	Real-time primer-Taqman
virB2-rev	CCTTTAATATTGGTAGTGTAGAACTAAGAGATTC	Real-time primer-Taqman
sodB1-for	CTGGAAAAGTCGCTGAAGCTATC	Real-time primer-Taqman
sodB1-rev	CGCTTTGAAATCGGCAAAG	Real-time primer-Taqman
rrsA-for	CACGATTACTAGCGATTCCGACTT	Real-time primer-Taqman
rrsA-rev	CGTCGTAGTCCGGATTGGA	Real-time primer-Taqman
virB 3′-for	GCGCGAAAGTCACTCGTC	Real-time primer-SYBR
virB 3′-rev	GAGATTCATTAGCCTTTTCAAGTCC	Real-time primer-SYBR
virB 5′-for	AACAGAAGAATCATTAGCCGATA	Real-time primer-SYBR
virB 5′-rev	TACGAGTGCCATCCAGAA	Real-time primer-SYBR
sodB-for	TCCGCTGCTGACCGTTGATG	Real-time primer-SYBR
sodB-rev	CGCCCAGAAGTGCTCCAGATAG	Real-time primer-SYBR
rrsA-for	AACGTCAATGAGCAAAGGTATTAA	Real-time primer-SYBR
rrsA-rev	TACGGGAGGCAGCAGTGG	Real-time primer-SYBR
MBG608	TCCAGAATTCAGTTGATTTGAC	Construction of pSRG12
MBG607	ACAAAAGCTTGAACATTGGGTCATATTACA	Construction of pSRG12
virB5	GCTCTAGAGCAAAAGATGTAACCACTTCCC	Construction of altered *virB*
virB6	GCTCTAGAGAACGAAGTCTGTTAATGGTGG	Construction of altered *virB*
virB12	GCATTCGTGAGTTGGGCATCGGCCTTAATTTTCTGAAAGTATCAGGG	Construction of altered *virB*
virB13	TAAGGCCGATGCCCAACTCACGAATGCTATGCTC	Construction of altered *virB*
sodB probe for	CTACTGGAACTGCCTGG	Amplification of northern blot probe
sodB probe rev	GTGCTCCAGATAGCCAG	Amplification of northern blot probe
**Probes**		
virB2	6FAMAGGACTTGAAAAGGCTMGBNFQ	Real-time probe-Taqman
sodB1	6FAMCCGCATCTTTTGGCMGBNFQ	Real-time probe-Taqman
rrsA	6FAMATGGAGTCGAGTTGCAGMGBNFQ	Real-time probe-Taqman
ssvirB-sense	TGCTCCTGCATATATTGCAGATGCTCTTCTACGAGTGCCAATTTCAATTCTACC ATCAATCTCCCTTCCTATTACAGGGAATTGTTGTAGC	Northern blot
ssvirB-antisense	TAGCATCCGAGAACTTGGTATTGGTCTTAATTTTCTGAAAGTATCAGGGATGTCCT ATAAAGACATAGCCAAAAAAGAGAATCTGTCTCGCGCGAAAGTC	Northern blot

### Site-directed Mutagenesis of *virB*

Specific nucleic acid modifications were introduced into *virB* by allelic exchange [21]. Briefly, splice overlap polymerase chain reaction (PCR) was used to amplify *virB* containing the desired nucleic acid changes and 500 bp of flanking sequence on either side [21]. Initial upstream and downstream products were amplified using oligonucleotides virB-5/virB-12 and virB-6/virB-13 respectively. PCR conditions: denaturation for 30 seconds at 95°C, annealing for 45 seconds at 50°C, and extension for 60 seconds at 72°C for 30 cycles in a Peltier thermal cycler (MJ Research, Watertown, MA). The final 2 kb product containing the desired nucleic acid changes, including an additional *Hae*III restriction endonuclease recognition site, was amplified with oligonucleotides virB-5 and virB-6. The resulting product was digested with *Xba*I and cloned into the *Xba*I site of pCVD442N2 to generate pERM124. The insert of pERM124 was sequenced to confirm that only the desired nucleic acid changes were incorporated. Osmotic shock was used to transform DH5α(*λpir*) with pERM124, and the plasmid was subsequently moved by conjugation into *S. dysenteriae* O-4576S1-G. Primary integrants were selected by growth in the presence of 200 µg/ml streptomycin and 250 µg/ml carbenicillin. Primary integrants were analyzed by PCR using oligonucleotide sets virB-1/virB-10 and virB-4/virB-9, followed by the purification of each product and digestion with the *Hae*III restriction endonuclease. Once confirmed, the selected primary integrant was cultured to mid-logarithmic phase in LB supplemented with 0.1% sucrose at 30°C. The culture was then serially diluted and plated onto LB agar plates containing 5% sucrose. The plates were incubated overnight at 37°C. Resulting colonies were screened for sensitivity to 250 µg/ml carbenicillin. Colonies sensitive to carbenicillin (8 of 120) were screened for the presence of the altered *virB* nucleic acid sequence by PCR using virB-4/virB-9 and virB-1/virB-10 oligonucleotide sets followed by digestion of each purified product with the *Hae*III restriction endonuclease. Sequence analysis was used to verify that only the desired nucleic acid changes were present in the resulting altered *virB* open reading frame.

### Real-time Polymerase Chain Reaction

RNA was isolated using RNeasy® Midi Kit (QIAGEN, Valencia, CA) per the product directions, from bacteria cultured for approximately 20 hours at 37°C on TSB agar plates supplemented with 0.1% Congo red, 250 µg/ml carbenicillin and 200 µM IPTG. Each RNA sample was then treated with 16 units of amplification grade DNaseI (Invitrogen, Carlsbad, CA), ethanol precipitated and dried. The RNA pellet was resuspended in DEPC-treated water, and the nucleic acid concentration was measured using an ND-1000 spectrophotometer (NanoDrop Technologies, Wilmington, DE). No more than 10 µg of total RNA was used to generate cDNA with the High Capacity cDNA Archival Kit (Applied Biosystems, Foster City, CA) per the product directions. Each cDNA sample was diluted 10 fold in water, and 5 µl was used as the template for each amplification reaction. All Taqman based reactions utilized TaqMan Universal Master Mix (Applied Biosystems, Foster City CA). Primers and FAM labeled minor grove binding probes were designed using Primer Express software (Applied Biosystems, Foster City, CA). All SYBR based reactions utilized iQ SYBR Green Supermix (Bio-Rad, Hercules, CA) and primers were designed using Beacon Designer 7 software (Premier Biosoft, Palo Alto, CA). Regardless of the chemistry, *rrs*A was used as the normalizer for each sample, and all values were calibrated to the value obtained from the given parental strain carrying the vector control plasmid using the ΔΔCt method [22]. All primer pairs and amplification conditions were validated by the inclusion of a standard curve on each reaction plate, from which efficiency was calculated. All reactions were run in a 7300 Real-Time PCR system (Applied Biosystems, Foster City, CA) under standard reaction conditions.

### Tissue Culture and *in vivo* Plaque Assays

Henle cell monolayers were cultured in 6 well polystyrene tissue culture plates (Corning Inc. Costar, Corning, NY) in Gibco Minimum Essential Medium (MEM) (Invitrogen Corp. Carlsbad, CA) supplemented with 10% fetal bovine serum (FBS), 2 mM glutamine and a 1 X final concentration of non-essential amino acids (Lonza, Switzerland). Plates were incubated at 37°C in an atmosphere of 5% CO_2_.

Plaque assays were performed as published with minor modifications [23]. Briefly, bacterial strains were grown overnight at 37°C on TSB agar plates containing the appropriate antibiotic. Ten colonies were used to inoculate a 3 ml LB culture containing 250 µg/ml carbenicillin, 0.1% DOC, and 200 µM IPTG. Cultures were grown to mid-logarithmic phase at 37°C. 10^4^ bacteria, diluted in phosphate buffered saline (PBS), were used to infect the Henle cell monolayer in each tissue culture well containing 2 ml MEM supplemented with 250 µg/ml carbenicillin and 200 µM IPTG. Plates were centrifuged for 10 minutes at 600 x g in a Centra GP8 (International Equipment Company, Needham Heights, MA). Plates were incubated at 37°C for 1.5 hours, washed with 2 ml PBS, then covered with a 2 ml overlay composed of MEM supplemented with 0.3% glucose, 250 µg/ml carbenicillin, 200 µM IPTG, and 20 µg/ml gentamicin to kill any bacteria that had not invaded into a eukaryotic cell. Following incubation for 72 hours at 37°C, the plates were washed with 2 ml PBS, stained with Wright-Giemsa stain (Camco, Ft. Lauderdale, FL), washed with distilled water, and air dried.

### Construction of the P*icsA-lacZ* Reporter Plasmid pSRG12

The P*icsA-lacZ* reporter plasmid pSRG12 is derived from the low-copy number, broad host range, *lac* expression vector pRW50 [24] and carries the *S. flexneri icsA* promoter region (597 bp upstream of the beginning of the *icsA* gene) on an *Eco*RI/*Hin*d III restriction fragment. This places the *lacZ* gene directly under the control of the *icsA* promoter.

### mRNA Stability Assay

Bacterial strains were grown on TSB agar plates containing 0.01% Congo red and 250 µg/ml carbenicillin. Following overnight incubation at 37°C, a single colony of each strain was used to inoculate a 3 ml culture of LB containing carbenicillin. Each culture was incubated overnight at 30°C in a shaking incubator. 250 µl of each overnight culture was used to inoculate a 25 ml LB culture containing carbenicillin and 0.1% DOC which was incubated at 37°C in a shaking incubator to an optical density (OD_650_) of 0.7. Next, 200 µM IPTG was added to each culture followed two minutes later by the addition of 250 µg/ml rifampicin. The time of rifampicin addition was designated t=0. At each time point, 1 ml of culture was removed and mixed with 250 µl of RNAlater (Ambion, Austin, TX) to preserve the mRNA profile of the sample. RNA isolation and Real-time PCR was performed using SYBR technology as detailed above.

### Northern Blot Analysis

Northern blots were performed using the Ambion Northern Max kit (Ambion, Austin, TX) per kit directions. No more than 4 µg of total RNA was loaded into the wells of a 0.8% agarose gel and BrightStar Biotinylated RNA Millenium Markers (Ambion, Austin, TX) were used as size standards. Blots were hybridized overnight with 10 pM of the indicated probe at 46°C or 42°C with either single stranded, biotin labeled DNA probe (IDT, Coralville, IA) or double stranded DNA probe labeled using the BrightStar Psoralen-Biotin Nonisotopic Labelling Kit (Ambion, Austin, TX), respectively. Blots were visualized using the BrightStar Biodetect Kit (Ambion, Austin, TX) per the directions.

### β-Galactosidase Assays

β-Galactosidase assays were performed as detailed previously [21]. Briefly, strains were grown overnight at 37°C on TSBA with Congo red containing 250 µg/ml ampicillin and 20 µg/ml tetracycline. Single colonies were selected and cultured in 3 ml LB with appropriate antibiotics in a shaking incubator overnight at 30°C. 130 µl of each culture was used to inoculate a 3 ml subculture in LB with antibiotics and 200 µM IPTG. Each subculture was incubated in a 37°C shaking incubator and grown to an OD_600_ between 0.3 and 0.6. The bacteria present in 1 ml of each culture were pelleted and resuspended in 1 ml Z buffer. 400 µl of the resuspended bacteria were then diluted 1∶1 in Z buffer. Bacteria were then permeabilized by the addition of 50 µl 0.1% sodium dodecyl sulfate (SDS) and 100 µl chloroform and mixed by vortexing on high for 10 sec. The permeabilized bacteria were incubated at 30°C for 15 min prior to the addition of 160 µl of ortho-Nitrophenyl-β-galactopyranoside (ONPG), diluted to 4 mg/ml in Z buffer. Each tube was mixed by vortexing on high for 5 seconds and incubated again at 30°C until a color change was observed. The time required for each reaction to change color was noted. Each reaction was stopped by the addition of 400 µl of 1 M Na_2_CO_3_. Tubes were centrifuged at maximum speed for 2 minutes and the optical density at both 550 nM (OD_550_) and 420 nM (OD_420_) was measured. A reaction containing all components except the bacterial culture was used as the negative control in the experiment.

## Results

### RyhB does not Influence VirF Activity

Previous studies have demonstrated that the production of *S. dysenteriae* RyhB results in a significant decrease in the steady state level of *virB* mRNA, but does not influence the steady state level of *virF* mRNA [6]. These findings indicate that RyhB-dependent regulation of *virB* expression is not mediated indirectly via inhibition of *virF* transcription or by the destabilization of the *virF* message [6], but do not rule out the possibility that RyhB-dependent regulation of *virB* expression is mediated indirectly via decreasing translational efficiency of the *virF* message, decreasing VirF protein levels or decreasing VirF activity. Since all three of these possibilities are likely to lower the activity of VirF in the cell, a necessary first step in characterizing the full impact of RyhB on the VirB/VirF regulon was to determine if RyhB influences the ability of VirF to function as a transcriptional activator.

VirF increases the activity of the *icsA* promoter [25], [26] (Figure 1), therefore to test the impact of RyhB production on the activity of VirF, the activity of the *icsA* promoter was measured in the presence or absence of increased RyhB production. Wild-type *S. dysenteriae* and *S. flexneri* were transformed with P*icsA-lacZ,* a reporter plasmid carrying the *icsA* promoter fused to an otherwise promoter-less *lacZ* gene [27], and the *ryhB* expressing plasmid p*ryhB* [6] or pQE, the empty vector control, were subsequently introduced. To ensure that RyhB production was being up-regulated from p*ryhB* in the presence of the inducer, a P*icsP-lacZ* fusion plasmid [20] was used as a control since RyhB has been shown to negatively regulate this promoter via its modulation of *virB* [6], [20].

**Figure 2:**
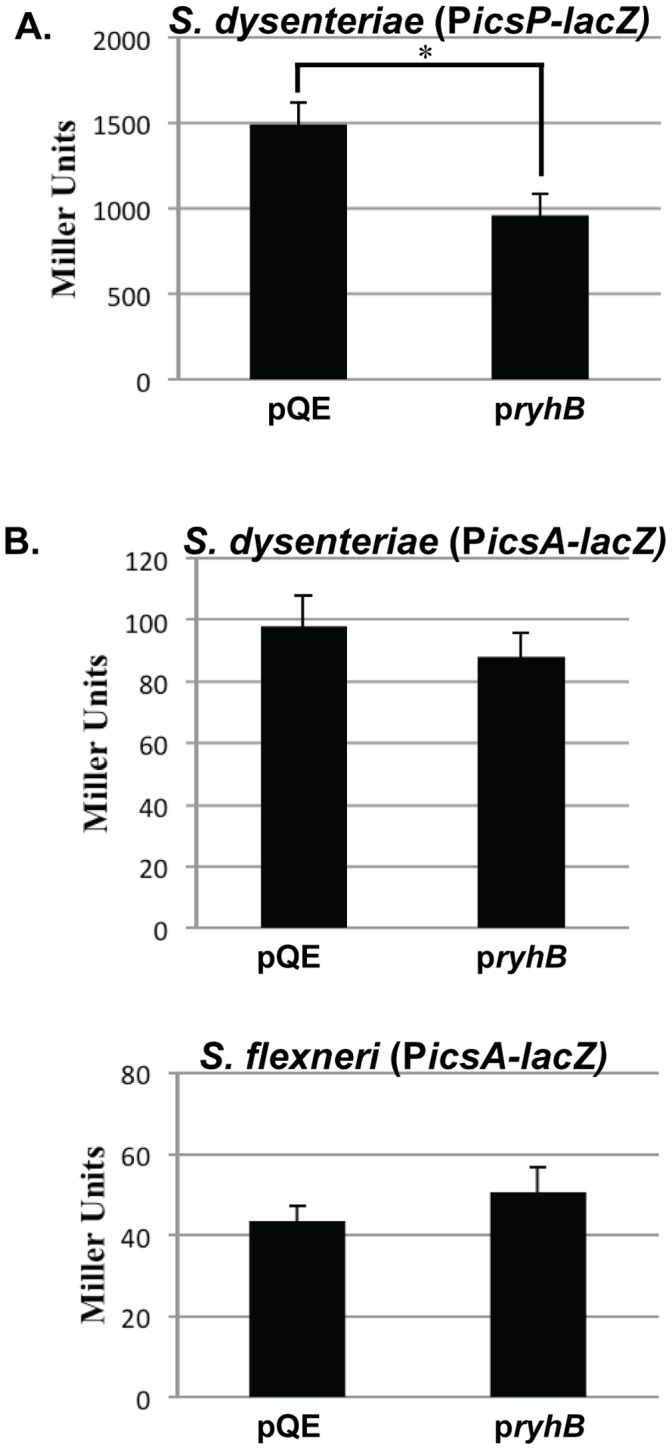
RyhB does not influence the activity of VirF. β-galactosidase activity measured in wild-type *S. dysenteriae* carrying P*icsP-lacZ* (**A**), and *S. dysenteriae* or *S. flexneri* carrying P*icsA-lacZ* (**B**), in both the presence (p*ryhB*) and absence (pQE2) of increased production of RyhB from the p*ryhB* plasmid. All cultures were grown in the presence of 200 µM IPTG to induce expression of *ryhB* from p*ryhB* when present. The data is the average of three independent experiments and error bars represent one standard deviation. *represents a significant difference from the activity of the strain carrying the pQE vector control (p≤0.01).

As seen previously in *S. flexneri* [20], production of RyhB from the p*ryhB* plasmid in *S. dysenteriae* resulted in a significant reduction in β-galactosidase activity produced from the P*icsP-lacZ* reporter (Figure 2A), indicating that the plasmid-based induction of *ryhB* was working as expected. In contrast, the activity of the P*icsA-lacZ* reporter in either *S. dysenteriae* or *S. flexneri* (Figure 2B) was not affected by the production of RyhB from the p*ryhB* plasmid. These data indicate that RyhB production does not influence *icsA* promoter activity. These findings strongly suggest that RyhB does not decrease the activity of VirF, the positive regulator of *icsA* promoter activity.

### RyhB Represses *virB* Transcription

The RyhB-dependent reduction of *virB* mRNA levels could result from lower levels of *virB* transcription, or from post-transcriptional regulation leading to the destabilization of the *virB* mRNA molecule. RyhB is known to down-regulate specific gene targets by facilitating destabilization of target mRNA molecules [28], [29], [30]. Furthermore, by a regulatory mechanism that is not yet fully characterized, stability of the *S. sonnei virB* mRNA molecule is altered in response to changes in environmental temperature, osmolarity and pH [14], [15], [16]. Based on these observations we next chose to determine whether or not RyhB production leads to destabilization of the *virB* mRNA molecule using *in vivo* mRNA stability assays and northern blot analyses. *sodB* mRNA was used as a control in each assay, as it is well established that RyhB functions to destabilize the *sodB* mRNA molecule [28], [31].

**Figure 3:**
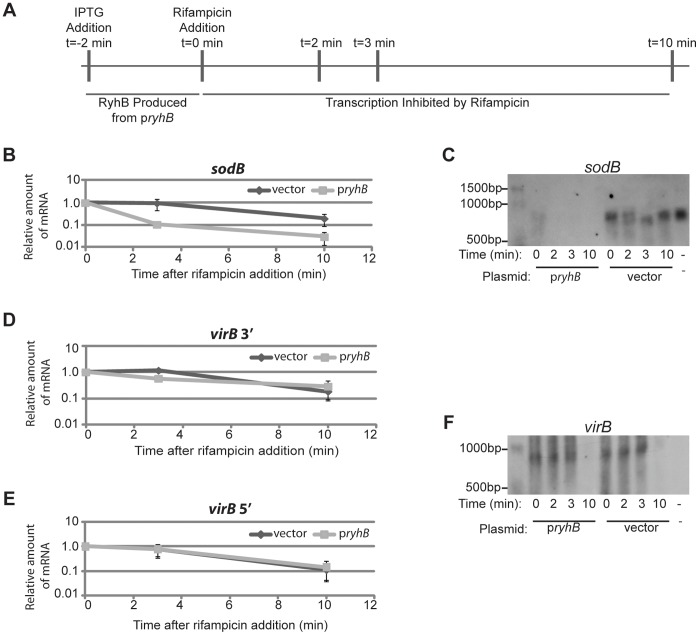
RyhB does not alter the stability of the *virB* mRNA molecule. Schematic of the procedure used to induce RyhB production, inhibit transcription, and collect RNA samples for Real-time PCR and northern blot based investigations into the impact of RyhB on the stability of *virB* and *sodB* mRNA molecules **(A).** The relative amount of *sodB* mRNA **(B)** and *virB* mRNA **(D** and **E)** was quantified by Real-time PCR in the presence (light lines) and absence (dark lines) of increased RyhB production. At each time point the amount of mRNA present is expressed relative to the level of mRNA at the time of transcriptional inhibition by the addition of rifampicin (t=0) and is normalized to the amount of *rrsA* measured in the sample. The data is an average of three independent experiments and error bars represent one standard deviation. The same set of RNA samples was used to measure levels of both *sodB* and *virB* mRNA. Northern blot analysis was used to detect full-length *sodB* and *virB* mRNA in each RNA sample collected (**C** and **F**, respectively). The negative control lane in each northern blot contains RNA isolated from wild-type *S. dysenteriae* carrying the pQE vector grown at 30°C, as *virB* expression is inhibited at this temperature. Each northern blot shown is representative of analysis performed with three biological replicates.

To investigate the effect of RyhB on the stability of *virB* mRNA and *sodB* mRNA, an mRNA decay assay was performed (Figure 3A). Briefly, wild-type *S. dysenteriae* carrying either p*ryhB* or the vector control was cultured to the mid-logarithmic phase of growth and IPTG added to induce production of RyhB from p*ryhB*. Two minutes after the addition of IPTG, transcription was halted by the addition of rifampicin. Samples were collected, and RNA isolated at the time of rifampicin addition (t=0) as well as 2, 3, and 10 minutes after rifampicin addition (t=2, 3 or 10). Relative amounts of *virB* mRNA and *sodB* mRNA were quantified by Real-time PCR. Analysis of *sodB* mRNA levels in the presence and absence of RyhB production demonstrated that the stability of *S. dysenteriae sodB* mRNA, like that of *E. coli sodB* mRNA [31], is dramatically reduced in the presence of RyhB (Figure 3B). These findings were confirmed by northern blot analysis, demonstrating that the production of RyhB from p*ryhB* dramatically accelerates the rate of *sodB* mRNA degradation as compared to the rate of *sodB* mRNA degradation in the absence of increased RyhB production (Figure 3C). The obvious reduction in the amount of *sodB* mRNA seen at t=0 in the strain carrying p*ryhB* as compared to that seen at the same time point in the strain carrying the vector control is to be expected given that RyhB causes the rapid degradation of *sodB* message, and that RyhB production was initiated two minutes prior to the collection of the sample (Figure 3A). These data confirm RyhB-dependent destabilization of the *S. dysenteriae sodB* mRNA and indicate that the *in vivo* assay is working as expected. Surprisingly, production of RyhB from the p*ryhB* plasmid had no effect on the stability of *virB* mRNA as determined by measuring the relative abundance of both the 3′ end (Figure 3D) and 5′ end (Figure 3E) of the message using quantitative Real-time PCR analysis. As above, these findings were confirmed by northern blot analysis (Figure 3F). Northern blot analysis of *virB* mRNA levels was performed using a single stranded DNA probe to eliminate the possibility that a transcript encoded anti-sense to *virB* was being detected (see Discussion). Together, these data clearly demonstrate that, unlike RyhB-dependent regulation of *sodB* mRNA, RyhB-dependent regulation of *virB* expression is not mediated by an increase in the rate of mRNA degradation.

Unlike that observed for *sodB* mRNA, *virB* mRNA levels were not dramatically different in the *S. dysenteriae* carrying the vector control as compared to the strain carrying the p*ryhB* plasmid at the point of transcriptional inhibition (t=0, Figure 3C and Figure 3F). This finding indicates that *virB* mRNA levels are not altered in the short time between the induction of RyhB production and the inhibition of transcription, further supporting the conclusion that RyhB does not function to destabilize *virB* mRNA.

RyhB-dependent reduction in the steady state level of *virB* mRNA, as seen in previous studies [6], together with the finding that this reduction is not mediated by destabilization of the transcript are consistent with RyhB regulating *virB* mRNA levels at the level of transcription.

### RyhB-dependent Regulation of *virB* Transcription is Facilitated by Nucleic acid Sequences within the *virB* Open Reading Frame

**Figure 4:**
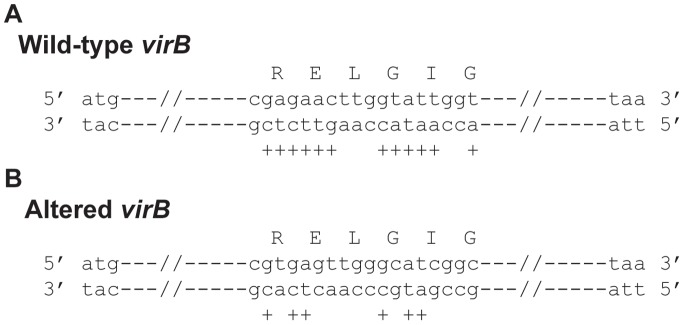
Nucleic acid complementarity exists between RyhB and the template DNA strand within *virB*. **(A)** A comparison of nucleic acid sequences within the wild-type *virB* gene to that within RyhB. The double stranded DNA sequence between base numbers 403 and 420 of 930 total bases in the wild-type *virB* gene is shown. The encoded amino acids are indicated using the single letter code. ";+"; indicates the location of nucleic acid sequence complementarity between the template strand DNA in the *virB* open reading frame and RyhB. **(B)** A comparison of nucleic acid sequences within the Altered *virB* gene to that within RyhB. The double stranded DNA sequence between base numbers 403 and 420 of 930 total bases in the Altered *virB* gene is shown. The encoded amino acids are indicated using the single letter code. Underlined sequences denote those that were mutated to construct Altered *virB.* ";+"; indicates the location of nucleic acid sequence complementarity between the template strand DNA in the Altered *virB* open reading frame and RyhB.

Each mechanism of direct RyhB-dependent regulation characterized to date has been shown to be mediated, at least in part, by nucleic acid complementarity between RyhB and the target mRNA molecule [28], [30], [32], [33]. The nucleic acid sequence of *virB* mRNA was examined for complementarity to RyhB, however no such complementarity was found. Instead, nucleic acid complementarity was observed between RyhB and the template DNA strand within the *virB* gene. Specifically, within the 930 nucleotide long *virB* coding sequence, twelve of eighteen nucleotides between base numbers 403 and 420 on the template DNA strand share perfect nucleic acid complementarity to nucleic acid sequences within RyhB (Figure 4A). Furthermore, five of the seven nucleotides between base numbers 414 and 420 of the *virB* gene share nucleic acid complementarity to five identically spaced nucleotides within the region of RyhB previously shown to mediate repression of *virB* expression [6] (Figure 4A).

**Figure 5:**
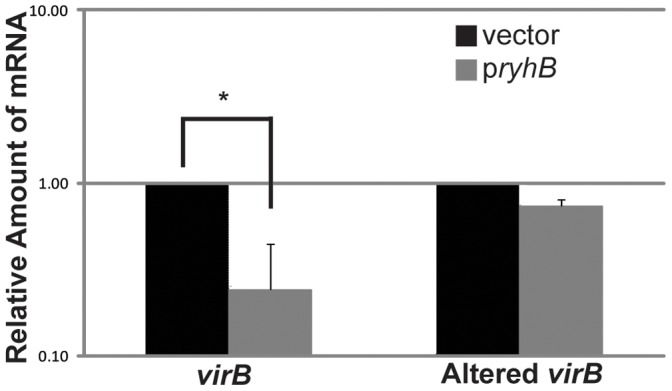
Alteration of nucleic acid sequences within *virB* reduces the efficiency of RyhB-mediated repression. Real-time PCR analysis of the amount of wild-type *virB* mRNA and Altered *virB* mRNA in the absence (vector) and presence (p*ryhB)* of *ryhB* expressed from an inducible plasmid promoter. The amount of mRNA is reported relative to the level of each in the strain carrying the vector control and is normalized to the amount of *rrsA* mRNA present in each sample. Each set of data is an average of three independent experiments. * denotes the existence of a significant difference between the indicated data points (p≤0.05).

The role of nucleic acid complementarity between RyhB and the DNA sequence of the template strand within the *virB* gene in mediating RyhB-dependent regulation of *virB* transcription was investigated by site-directed mutagenesis of *virB*. The nucleic acid mutations introduced into the *virB* gene, designated Altered *virB,* conserve the amino acid sequence of the encoded protein, but reduced the nucleic acid complementarity between *virB* and RyhB to six of the original twelve bases (Figure 4B). The impact of the site-directed mutagenesis on RyhB-mediated repression of *virB* expression was investigated using quantitative Real-time PCR. The relative amount of either wild-type or Altered *virB* was measured in the presence (p*ryhB*) or absence (vector) of increased production of RyhB. It is important to note that prior to normalization the level of Altered *virB* message was not significantly different than that of wild-type *virB* in each strain carry the vector control (data not shown). As seen previously [6] production of RyhB from the p*ryhB* plasmid resulted in a significant decrease in the steady state levels of wild-type *virB* mRNA, however production of RyhB had no significant effect on the steady state level of Altered *virB* mRNA (Figure 5). These data indicate that the identified nucleic acid sequence within the *virB* gene directly or indirectly facilitate RyhB-dependent regulation of *virB* expression.

### The Altered *virB* Mutant Displays Virulence-associated Phenotypes in *S. dysenteriae*, but the Phenotypes are not Affected by the Production of RyhB

**Figure 6:**
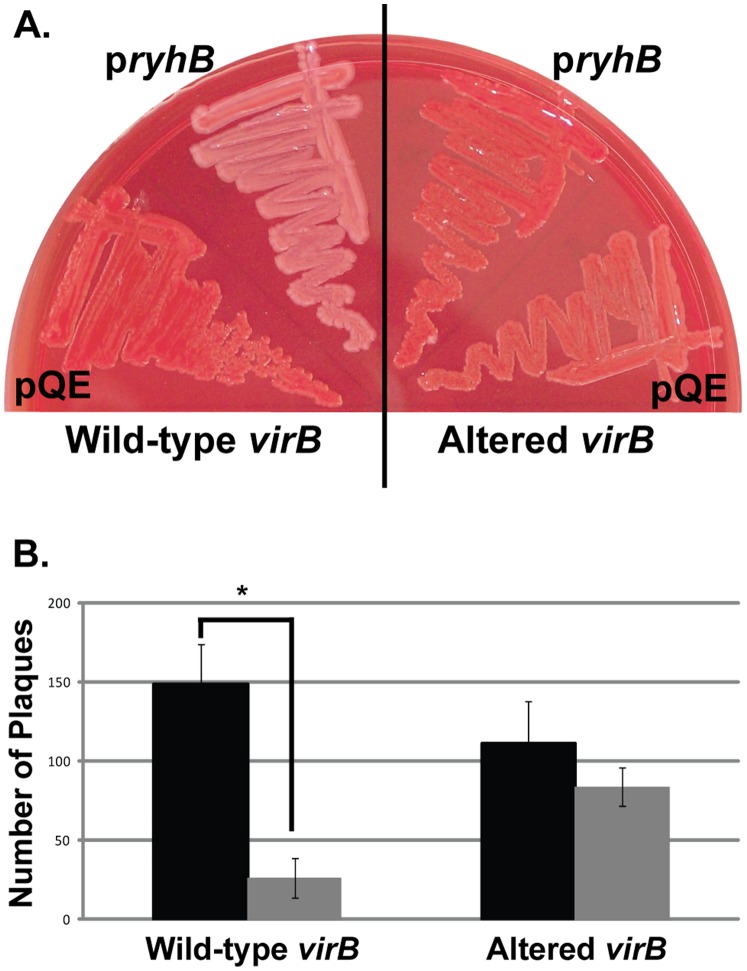
RyhB has no significant affect on VirB-dependent phenotypes in *S. dysenteriae* expressing Altered *virB.* **A.** Congo red binding by wild-type and Altered *virB* in *S. dysenteriae* in the presence (p*ryhB*) and absence (pQE2) of increased production of RyhB. All strains were cultured in the presence of 200 µM IPTG to induce expression of *ryhB* from p*ryhB*. **B.** Quantification of plaques formed by *S. dysenteriae* expressing wild-type (dark bar) or Altered *virB* (light bar) in the presence of *ryhB* expressed from the inducible plasmid promoter of pryhB and that measured in each strain carrying the vector control. All strains were cultured in the presence of 200 µM IPTG to induce expression of *ryhB* from p*ryhB.* Data represents the average of four independent experiments. *represents a significant difference from the number of plaques formed by the strain carrying the pQE vector control (p≤0.01).

*Shigella* virulence is positively correlated with the ability of the bacterium to bind Congo red from an agar medium [34] and this phenotype has been used previously to determine the effects of RyhB on production of VirB-dependent gene products [6]. Therefore we next tested the ability of RyhB to suppress Congo red binding by wild-type *S. dysenteriae* or an Altered *virB* mutant. Each strain carrying the pQE vector control, displayed a positive Congo red phenotype, strongly suggesting that the mutations introduced into the Altered *virB* mutant do not disrupt function of the encoded protein (Figure 6A). Strikingly, unlike Congo red binding by wild-type *S. dysenteriae,* which was dramatically reduced following production of RyhB from the p*ryhB* plasmid, Congo red binding by the Altered *virB* mutant was only slightly lower in the presence of increased RyhB production (Figure 6A).

The ability of *Shigella* to form plaques within a Henle cell monolayer is a quantifiable virulence-associated phenotype that is dependent on the activity of VirB [23]. As a means to evaluate the ability of RyhB to inhibit VirB activity, the impact of RyhB production on plaque formation by wild-type *S. dysenteriae* and the Altered *virB* mutant was measured. As demonstrated by the number of plaques formed by wild-type *S. dysenteriae* carrying the vector control (149+/−25) and the Altered *virB* mutant carrying the vector control (111+/−27), the nucleic acid changes introduced into the mutant did not significantly affect the ability of this strain to form plaques as compared to wild-type *S. dysenteriae* (p=0.149) (Figure 6B). Consistent with previous data [6], production of RyhB from the p*ryhB* plasmid significantly repressed plaque formation by wild-type *S. dysenteriae* as compared to the strain carrying the pQE vector control (p=0.002) (Figure 6B). In contrast, in the Altered *virB* mutant, increased production of RyhB had no significant effect on plaque formation, as compared to the strain carrying the pQE vector control (p=0.188).

Together, these data clearly demonstrate that alteration of nucleic acid sequences within *virB* that reduce the level of nucleic acid complementarity between RyhB and the DNA sequence of the template strand within *virB* decrease the ability of RyhB to inhibit each VirB-dependent virulence-associated phenotype investigated.

## Discussion

The VirF/VirB regulatory cascade plays a central role in *Shigella* virulence, coordinately regulating the production of several virulence factors in response to environmental conditions encountered by the pathogen during the course of a natural infection. The hierarchical regulation of the VirF and VirB regulons [1] and the observation that factors affecting the post-transcriptional regulation of *virB* mRNA also affect *virF* expression [1], [14], [15], [16] has suggested that the expression of the VirF and VirB regulons is linked. This study reveals that RyhB modulates *virB* transcription and that this regulation is independent of VirF, providing the first experimental evidence of differential regulation of genes within the VirF and VirB regulons.

**Figure 7:**
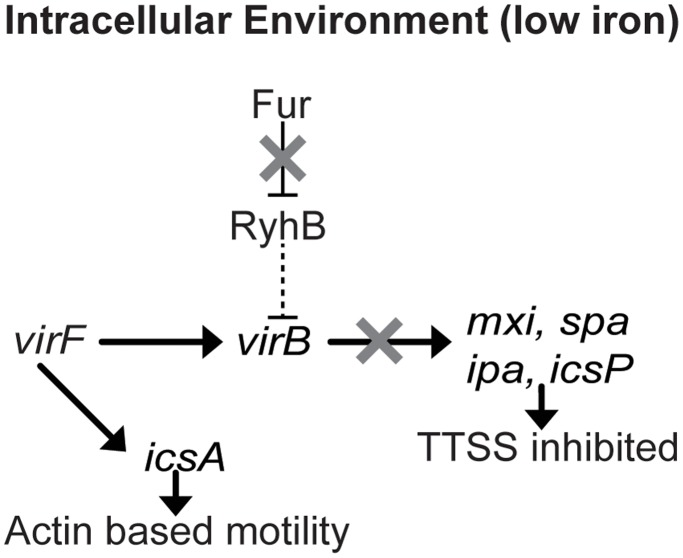
Working model of RyhB-dependent repression of *virB* transcription under iron poor conditions encountered within the intracellular environment. Under iron poor conditions, mimicking those likely encountered within the eukaryotic cell, Fur does not inhibit RyhB production and the RyhB functions to, directly or indirectly, inhibit *virB* transcription. Under such conditions RyhB-dependent VirF-independent regulation of *virB* expression facilitates the differential modulation of TTSS activity and actin based motility.

So far, three mechanisms of RyhB-dependent regulation have been characterized in *E. coli*. These mechanisms are exemplified by the regulation of *sodB,* the *isc* operon (not to be confused with the *Shigella ics* genes, also discussed in this work) and *shiA* [28], [29], [30], [31], [32], [33]. RyhB controls the expression of *E. coli sodB*, encoding superoxide dismutase, by binding to and ultimately destabilizing the *sodB* mRNA molecule [28], [29], [30]. Additionally, RyhB can facilitate differential gene expression from polycistronic mRNA molecules by altering the stability of a portion of the message, as has been demonstrated for the *isc* operon [33]. Finally, RyhB is predicted to promote the translational efficiency of *E. coli shiA* by binding to the 5′ untranslated region of the message and altering the structure such that an inhibitory element is eliminated [32]. Although the molecular mechanisms vary, common to each identified mechanism of RyhB-dependent regulation is the fact that regulation occurs following transcription and is dependent upon nucleic acid complementarity between RyhB and the target mRNA molecule.

As demonstrated in this study, several features of RyhB-mediated regulation of *virB* differentiate it from RyhB-dependent regulation of previously characterized gene targets. Unlike other targets of RyhB-mediated regulation that are chromosomally located, *virB* is encoded on a large virulence plasmid [4]. The regulation of a horizontally acquired, genus specific, virulence factor, by a conserved, chromosomally located, regulatory RNA, provides further evidence of the versatility of RyhB in controlling bacterial gene expression. The lack of nucleic acid complementarity between RyhB and the *virB* mRNA, as well as the presence of nucleic acid complementarity between RyhB and the template DNA strand within the *virB* gene distinguishes *virB* regulation from that of previously characterized RyhB targets.

The presence of nucleic acid complementarity between RyhB and template strand DNA within *virB* raises the possibility that RyhB is complementary to an mRNA or sRNA encoded anti-sense to *virB.* If present, the anti-sense transcript may play an intermediary role in facilitating RyhB-dependent regulation of *virB* transcription. In this study, the presence of such a transcript was investigated by northern blot using a single-stranded DNA probe with nucleic acid identity to *virB*. This analysis failed to detect a transcript anti-sense to *virB* in the region of the message with sequence similarity to RyhB (data not shown). These findings strongly suggest that RyhB affects *virB* mRNA levels via its complementarity with the template stand of *virB.*

While disruption of the nucleic acid complementarity between RyhB and the template strand of *virB* ( [6], Figure 4), reduces the efficiency of both RyhB-dependent regulation of *virB* expression (Figure 5) and RyhB-dependent modulation of virulence phenotypes (Figure 6), the precise role of these sequences in facilitating regulation remains unknown.

Finally, in contrast to previously characterized mechanisms of RyhB-dependent gene regulation, we have demonstrated that RyhB-dependent regulation of *virB* expression is mediated, directly or indirectly, at the level of *virB* transcription. Aside from RyhB and VirF, all previously identified factors shown to regulate *virB* transcription also influence *virF* expression. Thus, if RyhB-dependent regulation of *virB* transcription is indirect, it is mediated via the regulation of an as-of-yet unidentified transcriptional regulator. Future *in vitro* assays will explore the molecular mechanism underlying RyhB-dependent regulation of *virB* transcription.

In *Shigella* species, the RyhB-dependent regulation of VirB activity may allow the VirB regulon (genes encoding the TTSS) to be regulated, without affecting the expression of genes in the VirF regulon (*icsA,* which is required for actin-based motility). Independent regulation of the VirF and VirB regulons may be advantageous to the bacterium at specific times during an infection within the human host. Specifically, once within the intracellular environment *Shigella* must repress production of the TTSS in order to prevent premature lysis of the eukaryotic cell, while allowing the production of IcsA in order to facilitate intracellular spread. As transcription of *ryhB* is under control of the iron-responsive regulator Fur [6], [28], [35], and gene expression analysis has demonstrated that several Fur-regulated genes are expressed within the intracellular environment of epithelial cells [36], [37], [38], it is reasonable to expect that RyhB will be produced when *S. dysenteriae* is within this intracellular environment, as discussed in Africa *et al* [20]. Under these conditions, RyhB may function to inhibit *virB* transcription, thus reducing production of TTSS while simultaneously allowing VirF-activated transcription of *icsA* to proceed (Figure 7).

In conclusion, this study demonstrates that *virB* transcription can be uncoupled from the activity of VirF by RyhB, revealing a unique mechanism by which genes within the VirF and VirB regulons can be differentially regulated. These findings not only expand the current understanding of the regulatory circuit controlling *Shigella* virulence gene expression, but also highlight the versatility of RyhB in regulating bacterial gene expression.
